# Post traumatic stress symptoms, anxiety, and depression in patients after intensive care unit discharge – a longitudinal cohort study from a LMIC tertiary care centre

**DOI:** 10.1186/s12888-020-02632-x

**Published:** 2020-05-12

**Authors:** Swagata Tripathy, Swati P. Acharya, Santosh Singh, Suravi Patra, Biswa Ranjan Mishra, Nilamadhab Kar

**Affiliations:** 1 Department Anesthesia & Intensive Care, Bhubaneswar, India; 2 All India Institute of Medical Sciences, Bhubaneswar, India; 3 Department of Psychiatry, Bhubaneswar, India; 4 AIIMS, Bhubaneswar, India; 5 Black County Partnership NHS Foundation Trust, Wolverhampton, UK

**Keywords:** Anxiety, Depression, Low middle-income countries, Intensive care, Mental health, Posttraumatic stress symptoms, Quality of life, Outcomes, India

## Abstract

**Background:**

Data on intensive care unit (ICU) related psychiatric morbidity from Low Middle-Income Countries are sparse. We studied the ICU related posttraumatic stress symptoms (PTSS), anxiety, and depression symptoms in a cohort of patients from Eastern India.

**Methods:**

We included adults admitted more than 24 h to a mixed ICU. PTSS, anxiety, and depression symptoms were assessed by telephonic or face to face interviews by using the Impact of Events-r (IES-r) and Hospital anxiety and depression (HADS), respectively, at 0, 7,14, 30, 90 and 180 days from ICU discharge. The loss to follow up was minimal. Demographic, socioeconomic, quality of life (QOL), and critical care related variables were studied.

**Results:**

Of 527 patients, 322 (59.4%) completed 6 months’ follow up. The majority were male (60%), mechanically ventilated > 48 h (59.4%), mean age of 48 (+/− 16), mean acute physiology and chronic health evaluation II (APACHE II) at admission 9.4 (+/− 4.6), median length of stay 3 (2–28 days). The rates of ICU related clinical PTSS was < 1 and < 3% for anxiety/depression at any point of follow up. Data were analyzed by linear mixed (random effects) models. There was a significant drop in all scores and association with repeated measures over time. Poor QOL at discharge from the ICU showed significant association with PTSS, anxiety, and depression (β = − 2.94, − 1.34, − 0.7 respectively) when corrected for gender and education levels. Younger age, greater severity of illness, and prior stressful life experiences predicted worse PTSS (β = − 0.02, 0.08, 3.82, respectively). Benzodiazepines and lower sedation scores (better alertness) predicted lower depression symptoms. (β = − 0.43, 0.37 respectively).

**Conclusion:**

ICU related psychiatric morbidity rates in our population are low compared with reported rates in the literature. Poor QOL at ICU discharge may predict worse long-term mental health outcomes. Further research on the impact of ICU and sociocultural factors on mental health outcomes in patients from different backgrounds is needed. The study was registered at CTRI/2017/07/008959.

## Background

Admission to the intensive care unit (ICU) is a stressful experience. The mental health problems associated with ICU admission have been broadly reported as posttraumatic stress, anxiety, and depression [1]. Substantial research has been undertaken in Europe and North America to identify rates, associated risk factors and preventative or therapeutic interventions [2]. In these studies, the rates of psychiatric morbidity are high (up to 20% of survivors). They are associated with increased functional impairment, health care burden, costs, and worse quality of life (QOL) [1, 3]. There is, however, sparse data for post ICU mental health outcomes from the developing world -low, middle-income countries (LMIC).

It is well known that various factors influence mental health outcomes following a traumatic incidence. Besides the type and intensity of trauma (ICU related factors), factors like sociocultural affect, post-trauma support, coping strategies and psychosocial support available also contribute to the development of posttraumatic stress [4–6]. Besides health-related concerns, admission to the ICU may have worries regarding the risk of death, post ICU disabilities, and functioning.

We hypothesized that mental health after ICU admission might be affected by ICU related factors as well as various socioeconomic and cultural factors specific to our population, such as education, employment, marital status, having young children, family support, substance addiction, and stressful or adverse life experiences/events. We aimed to study the levels of ICU related posttraumatic stress symptoms (PTSS), anxiety and depression, and the factors contributing to these among ICU survivors. We expect that knowledge of the psychiatric morbidities and the factors influencing them may improve our understanding of the phenomenon, particularly in times when ICUs the world over are seeing patients from varying backgrounds. This may help in better management and patient outcomes.

## Methods

In this prospective observational study from January 2017 to July 2018 patients admitted to the ICU for more than 24 h, were assessed on the day of discharge from ICU (day 0) and then followed up at prefixed intervals of 7, 14, 30, 90 and 180 days to examine psychological outcomes of survivors. The institutional ethics committee approved the study protocol, and it was registered at the Clinical Trials Registry- India (CTRI) (CTRI/2017/07/008959). Written informed consent was obtained from all patients or next-of-kin for participation.

### Study center

The study was conducted in All India Institute of Medical Sciences, Bhubaneswar, a 600-bed tertiary level teaching hospital with an 8-bedded post-surgical and 17 bedded mixed ICU. Government funding enables subsidized cost to patients. The ICU areas have round the clock coverage by trained intensivists and nurses, with a nurse to patient ratio of 1:2–3. Liberal use of dexmedetomidine in place of benzodiazepine infusion and a protocol of morning sedation vacation is practiced. Physical restraint is used if the patient poses a danger to self or others, and other methods have been ineffective.

### Patients

**Fig. 1:**
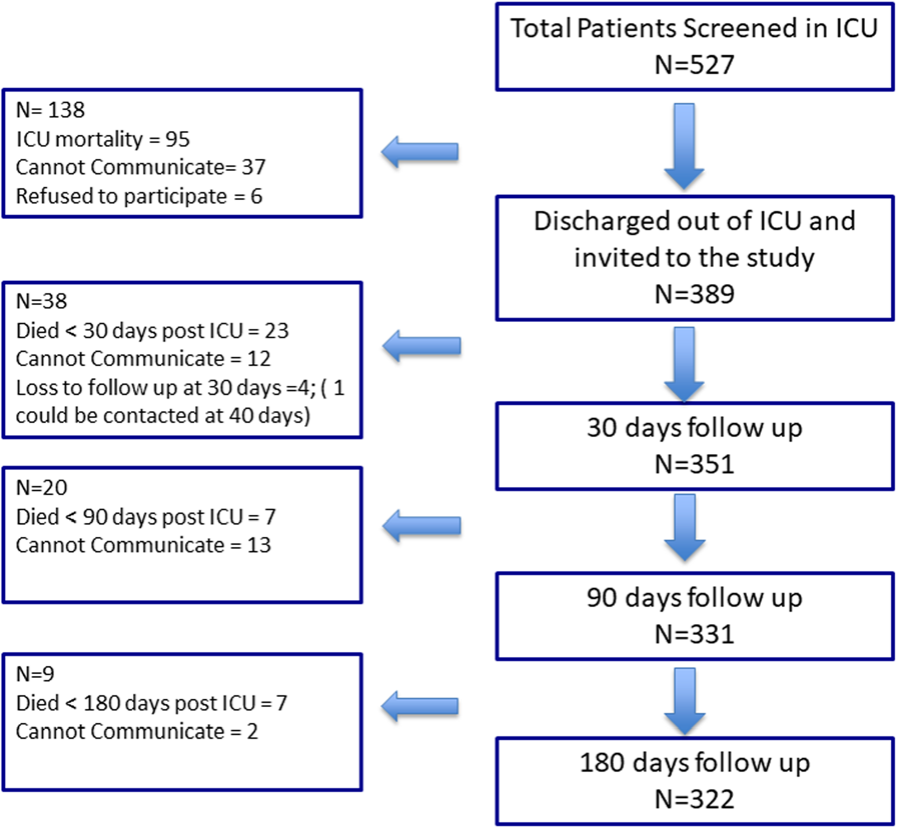
STROBE diagram showing the flow of patients in the study

Adult patients expected to stay for more than 24 h, survived to ICU discharge, and consented to participate (self or next of kin) were included (Fig. 1). If the patients were incapacitated, we took consent from the next of kin until they recovered. Death or inability to communicate meaningfully at discharge from ICU resulted in exclusion. Similarly, during follow-ups, patients who could not participate in the assessment due to being physically unwell with an inability to concentrate or difficulty in communication were also excluded. Readmission to the ICU within the same hospital stay was treated as a single event.

### Research team

The research team consisted of consultants and postgraduate trainees of the critical care team, consultants of the department of psychiatry, and a clinical psychologist (engaged full time for this study).

In the ICU and before discharge from the hospital, the clinical psychologist visited the patients, explained about the study, and completed consent procedures. On the day of discharge and on the 7th day, the clinical psychologist also supported the consenting patients in answering the self-rated mental health and QOL questionnaires. Follow-up interviews (on day 14, 30, 90, and 180) were conducted over the telephone; however, some were done when patients came for follow up to the hospital and at patients’ homes. None of the patients had only face-to-face or only-telephone interview. The telephone interviews were conducted at a suitable time for the patients; they were allowed to stop the call anytime and request a return call when they desired. In some instances where telephone interviews could not be held satisfactorily, e.g., the patient seemed uncomfortable to talk over the phone or requested a visit, home visits were made within a 150-km radius as per the provision of the research protocol and funds. (Supplement 1) Around 100 such interviews were done at home. The assessments done at home were aided by an audio recording to improve data capture for reference in case of any queries. The follow-up assessments were supported by a specific script and guidelines (Supplement 2).

All mental health related questionnaires were self-rated by the patients. The clinical psychologist in the research team supported patients in the process. If there were any clinical concerns or their responses seemed significant to the clinical psychologist, patients were offered consultation opportunities at the hospital with the psychiatrists in the team.

### Data collection and questionnaires

The team collected clinical variables during the ICU stay, including the reason for ICU admission, length of ICU stay, duration of mechanical ventilation, administration of corticosteroids, analgesic, paralytic and sedative drugs, pain, sedation, and delirium scores. We recorded medical history, including current substance abuse, prior/existing mental illness, previous admission to the ICU, exposure to stressful life-threatening events such as abuse, natural disaster, and accident. Demographic details such as education, employment status, having young children (less than 18 years’ age), and level of social support) were collected (Table 1). Acute Physiology and Chronic Health Evaluation (APACHE) II score [7], Sequential Organ Failure Assessment (SOFA score) [8] Charlson comorbidity index [9] and Richmond Agitation Sedation Score [10] were recorded for each patient.

**Table 1:** Sample characteristics

Variable	Total	Male (*n* = 192)	Female (*n* = 130)	*p* value
Age years mean (95% CI)	47.8 (46–49.5)	49 (47–51)	46 (43–49)	0.12
Education <6th grade n (%)	301 (93)	182 (94)	119 (92)	0.13
Married n (%)	288 (89)	169 (88)	49 (89)	0.31
Have young children < 18 yrs. n (%)	79 (25)	43 (22)	36 (28)	0.28
Employed n (%)	125 (39)	97 (51)	28 (22)	0.00
Substance Abuse n (%)	108 (34)	75 (39)	33 (25)	0.01
Experienced Life Stressors n (%)	6 (2)	2 (1.0)	4 (3)	0.18
Low Social Support n (%)	62 (19)	39 (20)	23 (18)	0.65
Indication for ICU stay n (%)		–	–	0.58
Postoperative	117 (36)			
Medical	101 (32)			
Neurological	68 (21)			
Trauma	36 (11)			
Length of Stay in ICU days mean (95% CI)	4.7 (4.2–5.1)	4.7 (4.1–5.2)	4.6 (4.0–5.2)	0.68
APACHE II mean (95% CI)	9.5 (9–10)	9.7 (9.0–10.4)	9.2 (8.4–9.9)	0.23
SOFA score mean (95% CI)	3.4 (3–3.7)	3.3 (2.9–3.7)	3.4 (2.8–4.0)	0.85
Mechanical Ventilation > 48 h n (%)	169 (53)	105 (54.7)	64 (50)	0.34
RAS scores mean (95% CI)	−0.4(− 0.3to-0.5)	−0.38 (− 0.24 to − 0.5)	−0.47 (− 0.33 to − 0.61)	0.38
Administered benzodiazepine n (%)	163 (51)	99 (51.6)	64 (49)	0.68
Administered Steroids n (%)	145 (45)	84 (44)	61 (47)	0.57
IESr in ICU median (IQR)	7 (6)	8(5)	7 (6)	0.16
IESr at 1-month median (IQR)	2 (2)	2 (2)	2 (3)	0.88
IESr at 3 months median (IQR)	0 (2)	0 (2)	0 (2)	0.87
HADS in ICU median (IQR)	10 (5)	10 (4)	8 (4)	0.71
HADS at 1-month median (IQR)	3 (2)	3 (2)	3 (3)	0.95
HADS at 3 months median (IQR)	2 (3)	3(3)	2 (4)	0.58
EQ5D VAS in ICU	35 (10)	35 (10)	35 (10)	0.3
EQ5D VAS 30 d	70 (15)	70 (11)	70 (15)	0.34
EQ5D VAS 90 d	80 (11)	80 (15)	80 (10)	0.69

*APACHE* Acute Physiology and Chronic Health Evaluation score, *SOFA* Sequential Organ Failure Assessment, *RAS* Richmond Agitation Sedation Scale, *IES-r* Impact of Events Scale-Revised, *HADS* Hospital Anxiety Depression Scale, *IQR* Inter Quartile Range, *EQ5D VAS* Visual Analogue Score in European Quality of Life 5 dimensions tool, *d* days

The Hospital Anxiety and Depression Score (HADS) [11], the Impact of Events Revised (IESr) [12] and European Quality of Life 5 Dimensions 3 levels (EQ5D- 3 L) [13] were administered to the patients at 0, 7, 14, 30, 90 and 180 days after discharge from the ICU. The HADS is a commonly used tool, which assesses levels of anxiety and depression. The questionnaire comprises 14 questions, seven each for anxiety and depression, interspersed within the questionnaire. These are scored separately. Cut-off scores used commonly are a score of 8 or more for anxiety (specificity of 0.78 and a sensitivity of 0.9), and depression (specificity of 0.79 and a sensitivity of 0.83). It takes around < 5 min to complete [11].

The IES-r has 22 questions for three subscales assessing for intrusion, avoidance, and hyper-arousal posttraumatic stress symptoms. Respondents are asked to identify a stressful life event (ICU stay in our cohort) and then indicate how much they were distressed during the past 7 days by each item listed. The cut-off scores for IES-r ≥ 30 [12].

EQ-5D is a standardized instrument for measuring general health status. Health status is measured in terms of five dimensions (5D); mobility, self-care, usual activities, pain/discomfort, and anxiety/depression. The EQ VAS records the patients’ assessment of his/ her health condition on a scale of 0 to 100, with 0 being the worse health condition imaginable to 100 being the best.

All these questionnaires and instruments have been validated in ICU patients [2, 14] The EQ5D- 3 L has been validated in the local language (Odia) previously [15]. A bilingual professional translated the HADS and IES-r from English to Odia. Another bilingual translator did the back translation according to the Brislin model for cross-cultural research [16].The Odia versions used in this study were pilot-tested on a discharged ICU patient who had consented to be in the study. We made final revisions after the pilot interview.

Multidimensional Scale of Perceived Social Support (MSPSS) was used to record the level of social support. It is designed to measure perceptions of support from family, friends, and a significant other. The scale is comprised of a total of 12 items, with four items for each subscale. Lower MSPS scores indicate lower social support. A mean scale score from 1 to 2.9 is considered low support, scores of 3 to 5 as moderate support, and 5.1 to 7 as high support [17].

The sample size was decided from previous studies in the Western world, assuming the proportion of patients having post ICU psychiatric morbidity to be 20% [2]. Considering a probability of Type 1 error of 0.05 and 80% power and to have at least 50 patients with 180 days follow up, we needed 310 patients. Counting for deaths and loss to follow up, we needed to recruit 510 patients.

### Analysis

Data were entered into an excel spreadsheet. We did a random cross-checking with data directly from the source case record forms at two time points (8 and 16 months), as a measure of quality control. The clinical psychologist’s presence in the interviews minimized missing data. Missing values were replaced with the mean of the rest of the item scores.

Results are expressed as mean ± standard deviation (SD) unless indicated otherwise. Numerical variables with ordered categories are described as the median and interquartile range (IQR). Student’s t-test was used for normally distributed continuous variables; Mann–Whitney U-test was used for variables in ordered categories and χ^2^ statistics, or Fisher’s exact test when appropriate, were used for categorical data. We assessed the mental health outcome scores in two ways. i) We divided the cohort into high or low symptom groups (those with scores>75th percentile of cohort scores and those with less) and analyzed predictors at 1 and 3 months of discharge. ii) We performed longitudinal mixed-effects regression analyses with the outcome scores as continuous variables to assess predictors over the entire follow-up. The linear mixed model has been recommended for repeated measures [18].

The ICU related and ICU non-related predictor variables were entered in the models as dictated by prior hypothesis and by significance (*p* < 0.1) in univariate analyses.

Statistical analyses were conducted using software packages (SPSS 25.0; SPSS Inc., Chicago, IL, USA and R Statistical Software v3.6.1 - R Core Team, 2019, Vienna, Austria).

## Results

Of 527 patients screened, 331 were recruited for the study, and 322 patients completed follow up at 6 months. (Fig. 1) Six were excluded for lack of consent, and four did not respond to calls at 1 month; of these, one was located at 40 days after letters were sent to his address and we accepted the data as for 1 month. The mean duration ±SD of follow up was 196 ± 7 (minimum 180) days.

Sixty percent of the participants (*n* = 192) were male. The mean age ± SD of the sample was 48 ± 16 years, with a range of 18 to 85 years. A considerable proportion (19%) had less than primary level education, 38% were employed, 25% had children below the age of 18 years. A considerable proportion of the sample (31%) reported current substance abuse at the time of ICU admission- the distribution being betel quid (47%), alcohol (25%), tobacco power or paste (as *gutkha* or *gudakhu*) (15%), cannabis or opioids (10%) and cigarette (< 1%). One in five had low social support at the time of admission. Less than 3% had a history of prior admission to the ICU, and 2% had a history of exposure to life-threatening situations. None had a history of psychiatric treatment. Mean APACHE II score ± SD at admission was 9.4 ± 4.6; Charlson’s comorbidity index ranged from 0 to 9; and the median length of ICU stay was 3 (2–28 days). About one-third (36.3%) of patients were admitted for postoperative care, followed by medical (31.3%), neurological (21.2%), and trauma (11.2%) related causes. More than half (52.3%) of the patients received mechanical ventilation (MV) for > 2 days. Sample characteristics are presented in Table 1.

### Mental health outcomes

Longitudinal analysis of the IESr and HADS scores suggested that there was a rapid drop in the total and domain wise IES-r and HADS scores within the first 14 days. Median and IQR scores in the anxiety and depression subscales and IES-r scores are detailed in Table 2. The proportion of patients with scores above the clinical cut off points for PTSS and anxiety-depression symptoms are reported in Table 3.

**Table 2:** Psychiatric morbidity scores over the study period

	Median (IQR) Scores					
**Days after ICU discharge**	**0**	**7**	**14**	**30**	**90**	**180**
**IES-r**						
Total IES-r	7 (6)	5 (4)	4 (3)	2 (3)	0 (2)	0 (0)
Intrusion	3 (2)	2 (2)	1 (1)	1 (1)	0 (1)	0 (0)
Hyperarousal	2 (3)	2 (2)	2 (2)	1 (2)	0 (1)	0 (0)
Avoidance	2 (2)	1 (2)	0 (1)	0 (0)	0 (0)	0 (0)
**HADS**						
Total HADS	10 (5)	6 (3)	4 (3)	3 (2)	2 (3)	0 (0)
Depression Domain	7 (3)	4 (3)	3 (2)	2 (3)	2 (2)	0 (0)
Anxiety Domain	3 (3)	2 (2)	1 (2)	1 (2)	0 (1)	0 (0)
**EQ5D-3 L**						
EQ5D Index	0.24 (0.2)	0.32 (0.4)	0.65 (0.)	0.72 (0.1)	1 (0.2)	1 (0)
VAS EQ5D	35 (10)	45 (10)	60 (15)	70 (15)	80 (15)	95 (5)

*IES-r* Impact of Events Scale-Revised, *HADS* Hospital Anxiety Depression Scale, *EQ5D* Euro Quality of Life 3 Dimensions, *VAS* Visual Analogue Scale, *IQR* Inter Quartile Range. Mentioned as median score (IQR)

**Table 3:** Domain wise point prevalence of mental health outcomes at various points of follow up. The numbers are in percentages

	Proportions above cut off point					
**Time period in days**	0	7	14	30	90	180
Total HADS score > 12	72	2.7	0.6	1.2	0.9	0
HADS Anxiety (subscale score > 7)	6.3	0.3	0	0	0.3	0
HADS Depression (subscale score > 7)	33.2	5.4	2.7	0.9	0.9	0
PTSS- IES-r (cut off score > 30)	0.6	0	0	0	0.3	0

Cut off for IES-r total is > 30, and for HADS anxiety and depression, it is > 7 [10, 11]

#### Post traumatic stress

Based on the cut-off score of 30, only 0.6% of patients were above that at the time of discharge (Day 0), later none qualified for this, except at 3 months it was 0.3% The median and 75% Interquartile score were much below the clinical cut off points, which suggested that there was hardly any significant posttraumatic stress disease (PTSD) in the sample studied.

#### Anxiety

At the initial assessment at discharge from ICU (Day 0), anxiety cut off 8 or above was present in 6.3%; on day 7, only 0.3% and at day 14 none were above cut off point.

At Day 0, there was no significant difference in sociodemographic and clinical parameters. However, a significantly higher proportion of patients in higher education group (> 5th grade) 28.6% vs. lower education group 3.2% (*p* < 0.001); those who did not have vasopressin 8.5% v 2.5% who had it (*p* < 0.5); those who received (10%) vs. did not receive benzodiazepines (2.5%) (*p* < 0.01); those who received (9.5%) vs. did not receive (3.8%) steroids (*p* < 0.05), had anxiety above cut off point.

#### Depression

On the day of discharge from ICU (Day 0), 33.2% had 8 or more in HADS-D. There was no difference in different sociodemographic or clinical variables studied, except for a few. Significantly more proportions of patients with a history of exposure to traumatic event (25%) vs with no such history (37.2%) (*p* < 0.05); patients with no history of drug abuse (37.1%) vs with drug abuse (25.2%) (*p* < 0.05); with septic shock (50%) vs without (30.5%) (*p* < 0.01) and who had sedation (47.2%) vs no sedation (25%) (*p* < 0.001) had depression.

#### Analysis of factors associated with mental health outcomes

In the first analyses, the patients were divided into a high vs. low symptom group (> 75th percentile score vs. < 75th percentile) for each of the three outcomes, and multivariate logistic regression models were set up at 1 and 3 months of discharge from the ICU. After correcting for baseline factors, higher scores or more significant symptoms of PTSS/ Anxiety & Depression were seen in those with substance abuse, comorbidities and worse APACHE scores, whereas those who were married, had good social support and better QOL had lesser symptoms (Table 4).

**Table 4:** Results of Logistic Regression for variables affecting mental health outcome scores at 1 and 3 months of ICU discharge

Variable	B	SE	Wald	df	Sig.	Odds Ratio	95% CI for OR UPPER	LOWER
**Logistic Regression for HADS scores at 1 month of ICU discharge**								
Married	−1.01	0.43	4.42	1	0.04	0.36	0.14	0.93
Substance abuse	1.52	0.41	13.5	1	< 0.005	4.57	2.03	10.28
Social Support	−0.76	0.37	3.59	1	0.04	0.5	0.22	0.9
QOL score at 1 month	−4.21	1.09	14.5	1	< 0.005	0.015	0.002	0.131
**Logistic Regression for HADS scores at 3 months of ICU discharge**								
Substance abuse	0.93	0.41	5.3	1	0.02	2.5	1.15	5.68
Comorbidity score	0.67	0.26	6.5	1	0.01	1.9	1.17	3.3
Social Support	- 0.8	0.4	4.01	1	0.04	0.45	0.2	0.9
**Logistic Regression for IES-r scores at 1 month of ICU discharge**								
Substance Abuse	0.64	0.27	5.79	1	0.02	1.92	1.13	3.27
APACHE II score	0.06	0.03	4.17	1	0.04	1.06	1.002	1.12
QOL score at 1 month	−3.16	0.02	11.85	1	0.001	0.04	0.007	0.25
**Logistic Regression for IES-r scores at 3 months of ICU discharge**								
Substance abuse	0.73	0.28	6.59	1	0.01	2.08	1.19	3.65
Social Support	−0.86	0.37	5.49	1	0.02	0.42	0.2	0.86

Social Support – Based on scores on the MSPS scale, lower scores indicate less social support. QOL: Quality of Life based on EQ5D- EQ5D 3 L index scores where lower scores indicate worse QOL; Comorbidity score is the Chalrson comorbidity index, with higher scores indicating more significant comorbidity. APACHE II- Acute Physiology and Chronic Health Evaluation scores where lower scores indicate greater severity of illness in the first 24 h of ICU admission; HADS – Hospital Anxiety Depression scale; IES-r – Impact of events scale revised- indicative of symptoms of posttraumatic stress

Due to the low rates of ‘caseness’ in our patients and the nature of the data with repeated measurements over time, analyses were then done by linear mixed models. We tested several models. The unconditional model to examine mean differences in outcome variables across individuals without regard to time suggested that about 18, 20, and 1% of the total variation in the IESr, HADS A, and HADS D scores respectively, was due to inter-individual differences. We ran the models with time to explore change over time (significant with *p* < 0.01 for all three outcomes) and then separately with the ICU related and ICU non-related predictor variables as dictated by prior hypothesis and significance in univariate analyses. Two covariance structure models were generated to assess the error covariance structure of the longitudinal data. The model selection was based on the least Bayesian Information Criterion (BIC), and nonsignificant covariates were excluded. The Maximum likelihood method was used as we focused on fixed and random effects. The significant results for the three outcome variables- IESr, HADS-A, and HADS-D, when corrected for baseline factors like age, gender, and education are shown in Table 5. Details of the final models used, along with the standard coefficients table are in Supplement 3.

**Table 5:** Estimated fixed effects of predictors of mental health outcome scores

Parameter	Estimate	df	t	P	95% CI		
					Lower	Upper	
**IESr Scores**							
**Intercept**	9.11	338.09	2.65	< 0.01	7.87	10.36	
**Age**	−0.02	316.87	−2.38	0.02	−0.04	− 0.00	
**EQ5D**	−2.94	317.18	−4.15	< 0.01	−4.32	−1.55	
**Life Stressor (1)**	3.82	316.76	4.37	< 0.01	2.11	5.53	
**APACHE II score**	0.08	317.87	2.61	0.01	0.02	0.15	
**Time 2**	−2.24	1643.18	−12.79	< 0.01	− 2.59	− 1.9	
**Time 3**	−3.66	1643.18	−20.89	< 0.01	−4.01	−3.32	
**Time 4**	−5.32	1643.18	−30.33	< 0.01	−5.66	−4.97	
**Time 5**	−6.43	1643.18	−36.69	< 0.01	−6.78	−6.09	
**Time 6**	−7.24	1643.18	−41.06	< 0.01	−7.59	− 6.90	
**HADS A Scores**							
**Intercept**	3.76	337.9	12.45	< 0.01	3.17	4.35	
**EQ5D**	−1.34	315.97	−4.08	< 0.01	− 1.98	−0.69	
**Time 2**	−1.2	1643.03	−13.98	< 0.01	− 1.36	− 1.03	
**Time 3**	− 1.74	1643.03	−20.37	< 0.01	− 1.91	− 1.58	
**Time 4**	−2.07	1643.03	−24.19	< 0.01	− 2.24	− 1.9	
**Time 5**	− 2.4	1643.03	− 28.04	< 0.01	− 2.57	− 2.23	
**Time 6**	−3.03	1643.03	−35.20	< 0.01	−3.2	− 2.86	
**HADS D Scores**							
**Intercept**	6.79	356.40	22.8	< 0.01	6.21	7.38	
**EQ5D**	−0.7	317.5	−1.94	0.05	− 1.4	0.00	
**Benzodiazepines (1)**	−0.43	317.09	−3.36	< 0.01	−0.68	−0.18	
**Sedation (1)**	0.37	316.97	2.79	0.01	0.11	0.63	
**Time 2**	−1.89	1642.43	− 17.19	< 0.01	−2.11	− 1.68	
**Time 3**	−3.02	1642.43	−27.39	< 0.01	−3.23	− 2.8	
**Time 4**	−3.98	1642.43	−36.11	< 0.01	−4.19	− 3.76	
**Time 5**	−4.73	1642.43	−42.89	< 0.01	− 4.94	− 4.51	
**Time 6**	−6.3	1642.43	−56.82	< 0.01	−6.52	− 6.08	

EQ5D- EQ5D 3 L index scores, Life Stressor (1)- Stressful life situations present, APACHE II- Acute Physiology and Chronic Health Evaluation scores, Time 2–6- Scores at 7,14,30,90 and 180 days, Benzodiazepines (1)- received benzodiazepines during ICU stay, Sedation (1)- had a mean sedation score < −1 or > 1 on the Richmond Agitation Sedation Scale (signifying over or under sedation respectively)

In the final models, poor QOL scores at patients’ discharge from the ICU had a significant association with worse mental health outcomes of PTSS, anxiety, and depression over 6 months follow up - standardized β coefficients and 95% CI of − 0.50 (− 0.74 to − 0.27), − 0.23 (− 0.34 to − 0.12) and − 0.12 (− 0.24 to - 0.01) respectively. Younger age, exposure to stressful life experiences, and greater severity of illness (APACHE II Score) at admission to ICU were other independent predictors of higher PTSS. Having poor sedation scores and not having had benzodiazepines administered during the ICU stay increased the risk of depression.

## Discussion

We believe ours to be the first longitudinal prospective follow up from a LMIC about PTSS, anxiety, and depression. The results suggest that almost one-third of patients have clinically significant depression after discharge from ICU. However, the rates fall quickly in the first couple of weeks to 2.7%. Compared to depression, the anxiety symptoms were observed to be considerably less (6.3%), and only a few patients remain above the clinically meaningful score that after a week. The median score of the sample for IES-r was considerably below the cut-off score for the possibility of PTSD, which only a few patients (0.6%) had at the time of discharge from ICU, and none after that, except one at 3 months. This indicated that PTSD is uncommon in the studied sample from Eastern India.

Anxiety and depression- The rates of anxiety and depression The rates of anxiety and depression reported in previous studies in ICU patients from the Western world [2, 19, 20] vary widely depending on the screening tools used and the time points of follow up [2]. Previous studies that have used the HADS tool like us, (cut off score of 8) have found higher rates of anxiety than depression (43% vs. 30%) [19]. A cut off of 11 has resulted in lower rates of 18 and 11%, respectively [21].

PTSS- Among the publications which have used IES-r for PTSS screening like us, cut off scores have differed (range 18 to 33), follow up times have ranged from 1 month to greater than 2 years and the point prevalence of PTSS has varied from > 20% [22, 23] to < 10% [24, 25]. However, none of the studies reported such low proportions, as observed in this study. Similar to us, most others observe a decrease in these scores with time [2]. Although the populations in different studies may not be comparable, the lower proportion of patients having PTSS may be due to various possibilities. It may be possible that ICU experience was not perceived as traumatic, or the experience did not lead to PTSS, or culturally patients expressed more depressive symptoms than PTSS.

There may be further explanations about the low rates of psychiatric morbidity as compared to the western data. Firstly, determinants of resilience and skills needed to deal with a stressor/trauma in one community successfully may differ from those in another [26]. Survivors in our cohort demonstrated a high degree of acceptance of the discomfort of injections, bed care and physical restraints in a matter-of-fact way. There was a feeling that “all that could have been done was done.” We found a recurring sentiment of gratitude that they had survived while others’ around them had not. Lower expectations of health care among the patients in LMIC countries could be an explanation for the higher satisfaction rates in ICUs, which are better equipped and have more staff than the wards. Similar findings have been reported recently from other ICUs in LMICs in South Asia- a multicentric study involving 32 state ICUs a majority of ICU survivors found the ICU environment to be safe and calm and reported high levels of satisfaction (84 and 94% respectively). Importantly, specific ICU experiences that were recalled were reported as relatively stress-free [27].

Secondly, factors like religious and cultural beliefs regarding critical illness may have played a role. There have been concerns that questionnaires may not be valid across cultures even after careful adaptations [28].This will need further exploration and may have a broader implication for non LMIC countries which see an ever-increasing ICU load of patients of mixed cultural backgrounds [27–29].

Third, the methodology, constellation of symptoms, and relative contribution of ICU treatment towards post-ICU psychiatric outcomes in prior research have also been questioned in recent studies [30–32]. It is possible that the postal questionnaires used in many of the studies [2] led to a deviation from reporting about ‘ICU experience/ ICU relatedness’ at long term follow up. In other words, people responded about their general levels of stress, rather than that related to their ICU experience- the ‘trauma’ that the studies are trying to explore. A psychiatrist or clinical psychologist intimately involved in the interviews (and home visits as in our study) might circumvent this deviation. Also, an external comparison group was absent in a majority of previous studies (including ours); comparing with mental health outcome statistics of the general population may not be ideal for a cohort of post ICU patients.

Furthermore, post ICU mental illness may present differently from other traumatic experiences. Depression may have more somatic manifestations, explaining the association with the patients’ QOL at the time of reporting [33]. PTSS may present as a preoccupation with the reoccurrence of disease or fear of medical appointments [26, 33]. We observed similar behavior; many of our patients contacted the research team asking for over-the-phone prescriptions, expressing hesitation to revisit the hospital for non ICU related follow-ups. Contributing factors.

We found that lower age, previous stressful life experiences, and worse QOL at discharge from the ICU were independently associated with higher PTSS scores. There was no association with baseline factors like gender and education. All scores decreased significantly over time. APACHE II score on admission affected PTSS in our patients. This is reported infrequently in prior ICU studies, even though trauma severity has consistently been associated with the development of PTSD in broader PTSD literature [34]. The lack of association of APACHE II scores in prior ICU studies has been suggested to be due to problems related to the measurement of the construct [30, 35]. In-ICU risk factors like steroids, delirium, length of stay in ICU, and mechanical ventilation except for the use of benzodiazepines and sedation in ICU (which were independent predictors for depression scores) were also not found to be associated with the outcomes. This is similar to the findings in the meta-analysis by Parker et al. [2]

Allowing for repeated measurements over time in the linear mixed models approach did not reveal any significant association with sociodemographic factors contrary to our hypothesis. An independent association of low EQ5D scores with poor mental health outcomes, however, was retained, as reported in other studies [14, 36–38].

### Strengths

We have tried to address methodological problems identified with previous research in this area (absence of true prospective data, sampling bias, loss to follow up, non-response bias, and inadequate coverage of sociocultural factors during assessment) [31]. The study is prospective in its design and has an adequate sample size. It has a robust mechanism for follow up, which included home visits leading to very low attrition over the study period. It used a range of measures in the local language. The measures selected have prior validation in this population [39]. As the study was done in a tertiary level set up, which is a referral center in the region with no or minimal cost to the patients, the range of conditions treated was wide. We had an inclusive sampling strategy and included sociocultural information such as children < 18 years, marital status, and social support.

### Limitations

There are a few limitations to this study. Although we intended to measure delirium, we had to remove it from our final analysis due to less than ideal implementation of the CAM-ICU tool, which had been recently introduced on the unit. The apparent low APACHE II scores in our cohort (compared to previous studies) may be due to a strict triage criterion and an active ICU outreach team policy. In essence, day 1 APACHE II scores (reported here) usually deteriorate through the stay before improving. The study was not designed as a multicentric study, thereby limiting its generalizability to other ICU setups. External validity for research with psychology-based outcomes is difficult to claim. However, results with similar trends are being reported from this part of the world more frequently [26, 40]. We studied only the anxiety, depression, and posttraumatic stress as outcome measures; although common, there may be other psychiatric manifestations and presentations. With low psychiatric morbidity observed, it might be challenging to study the predictive potential of the contributing factors. While psychiatrists in the team reviewed the patients demonstrating high scores, a detailed psychiatric examination of each patient was beyond the scope of this study. There may be various patient, clinician, and other factors contributing to the mental health outcome; these were not studied.

## Conclusion

Considerable proportions of patients had clinically meaningful depressive symptoms at the time of discharge from ICU, although the rates decreased fast in a couple of weeks. Fewer patients had anxiety, which also improved quickly. Rates of PTSS were considerably low at the time of discharge and were subclinical. A probable reason for a lower proportion of PTSS in an Indian or Asian context could be lower stress perception, support received during ICU admission in a tertiary level of care with high reliability, and greater resilience in the population; however, these factors need a focused study. It is essential to highlight that these psychiatric morbidities, which might be affecting the recovery process, are often not recognized. A simple measure of screening, such as the QOL at ICU discharge, may be used for patients following ICU discharge. There is a great need for more studies about the mental health outcomes of ICU patients and their management in LMICs.

## Supplementary information

**Additional file 1.** Supplement 1- Geographic distribution of home visits made and some representative photographs.

**Additional file 2.** Supplement 2- Interview Guide.

**Additional file 3.** Supplement 3- Linear mixed models- statistics.

## Data Availability

The datasets used and analyzed during the current study available from the corresponding author on reasonable request.

## Abbreviations

ICU: Intensive care unit
QOL: Quality of life
PTSS: Post traumatic stress symptoms
IES-r: Impact of events-r
HADS: Hospital Anxiety and Depression Scale
LMIC: Low middle-income countries
CTRI: Clinical Trials Registry- India
APACHE: Acute Physiology and Chronic Health Evaluation
SOFA score: II score, Sequential Organ Failure Assessment
EQ5D- 3L: European Quality of Life 5 Dimensions 3 levels
IQR: Interquartile range
OR: Odds Ratio
