# Supplementary material for: Post traumatic stress symptoms, anxiety, and depression in patients after intensive care unit discharge – a longitudinal cohort study from a LMIC tertiary care centre
Source: BMC Psychiatry. 2020 May 12;20:220. doi: 10.1186/s12888-020-02632-x (PMC7216410; doi:10.1186/s12888-020-02632-x)
Supplement: Supplementary file 1 — Additional file 1. Supplement 1- Geographic distribution of home visits made and some representative photographs. [file 12888_2020_2632_MOESM1_ESM.pdf]

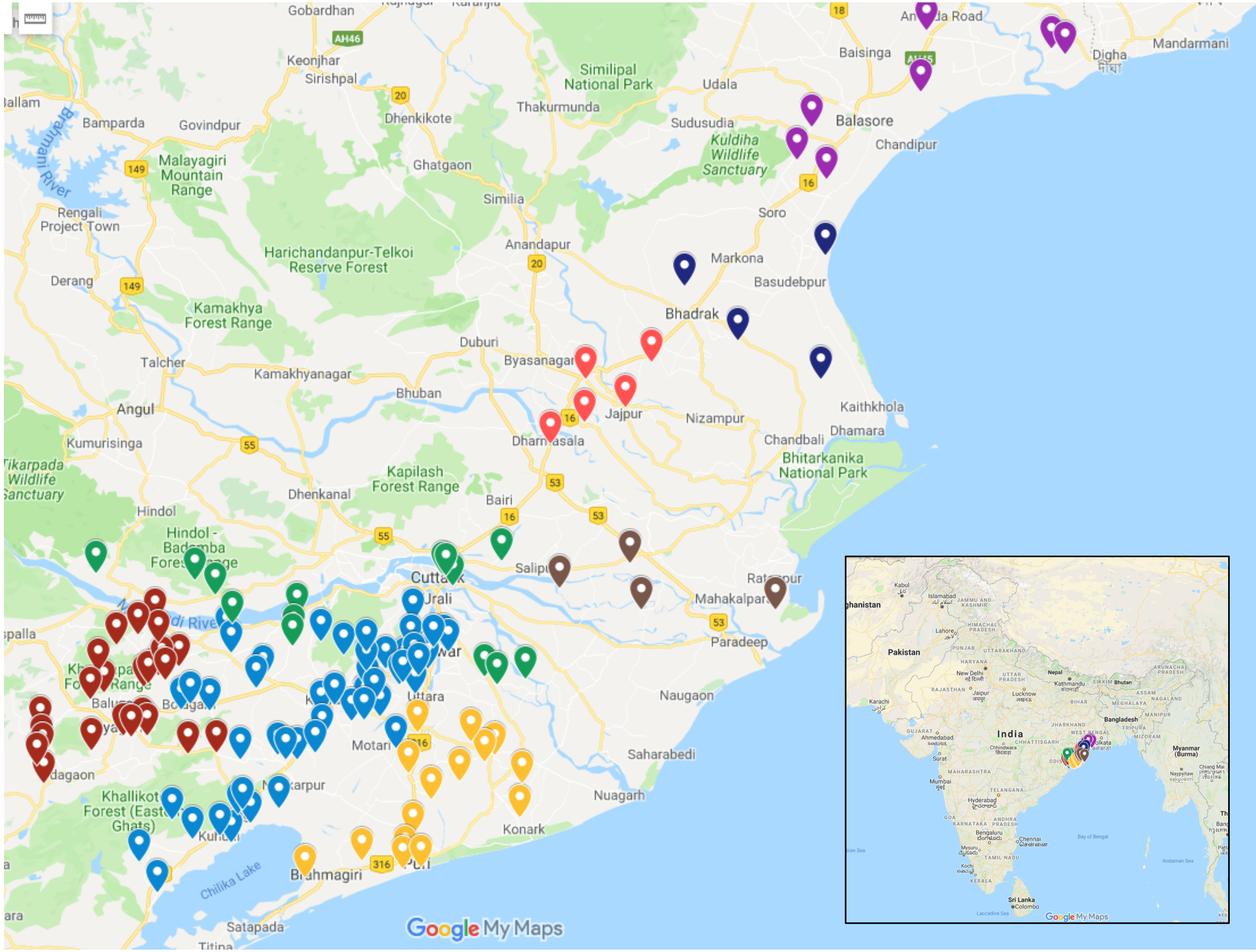

Fig.2 Location of home visits made for post ICU follow up. Courtesy "Google Map data: Google, DigitalGlobe". Google. (n.d.). [Google Maps depicting ICMO study phase 1 home visits]. Retrieved from; https://drive.google.com/open?id=1_Z82WIMl3wGYOxz2ISu8Nvhu5RGMFQku&usp=sharing

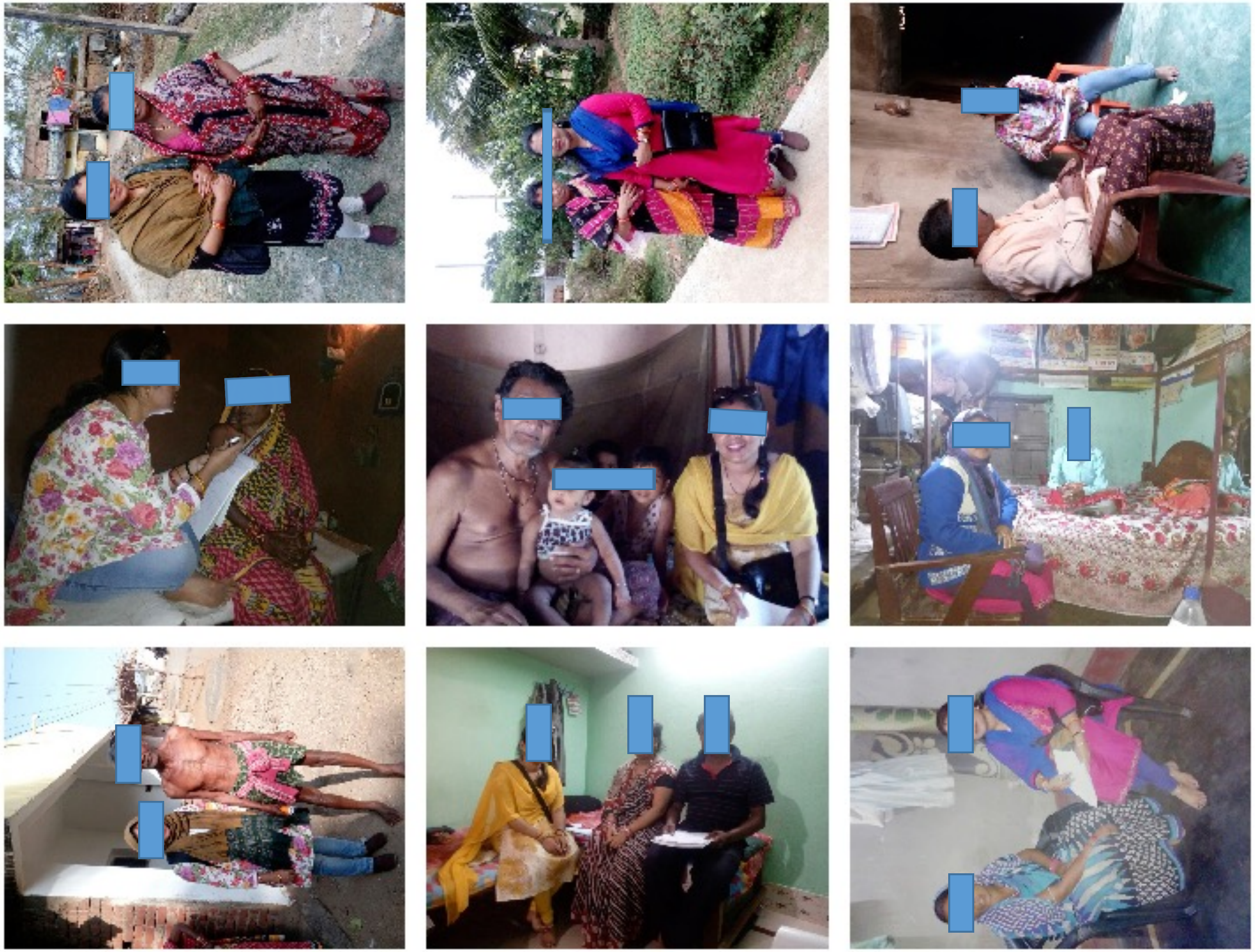

Representative photographs of Home Visits- All patients and their families have given written consent to be photographed and for their photographs to be used for academic purpose.
