# Supplementary figures and images for: Post traumatic stress symptoms, anxiety, and depression in patients after intensive care unit discharge – a longitudinal cohort study from a LMIC tertiary care centre
Source: BMC Psychiatry. 2020 May 12;20:220. doi: 10.1186/s12888-020-02632-x (PMC7216410; doi:10.1186/s12888-020-02632-x)

## Slide 1
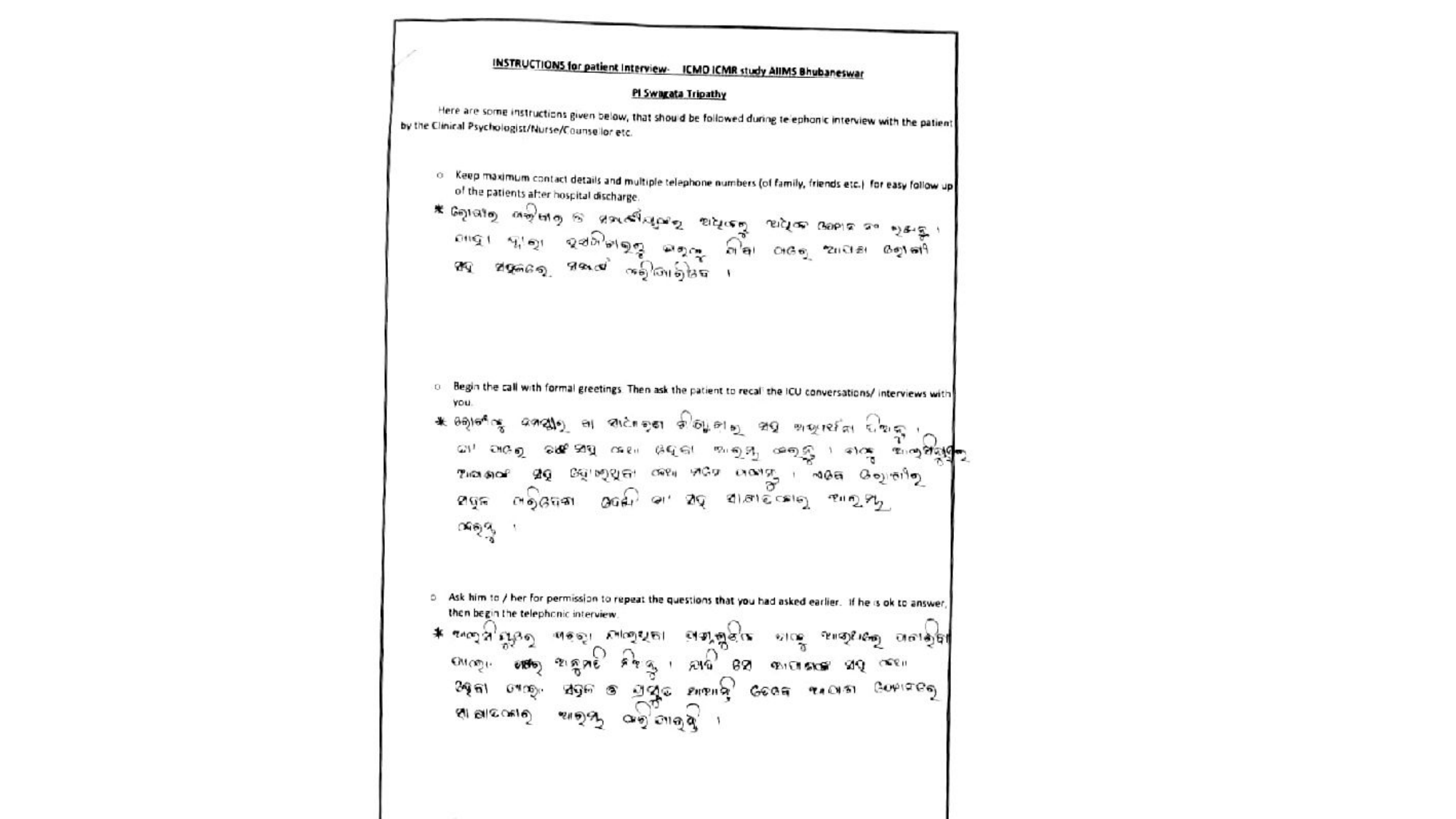

#

Supplement: Supplementary file 2 — Additional file 2. Supplement 2- Interview Guide. [file 12888_2020_2632_MOESM2_ESM.pptx]
