# Supplementary material for: Post traumatic stress symptoms, anxiety, and depression in patients after intensive care unit discharge – a longitudinal cohort study from a LMIC tertiary care centre
Source: BMC Psychiatry. 2020 May 12;20:220. doi: 10.1186/s12888-020-02632-x (PMC7216410; doi:10.1186/s12888-020-02632-x)
Supplement: Supplementary file 3 — Additional file 3. Supplement 3- Linear mixed models- statistics. [file 12888_2020_2632_MOESM3_ESM.pdf]

MODEL INFO:
Observations: 1979
Dependent Variable: ies_r_complete ICU
Type: Mixed effects linear regression

MODEL FIT:
AIC = 9414.77, BIC = 9532.17
Pseudo-R^2 (fixed effects) = 0.46
Pseudo-R^2 (total) = 0.67

FIXED EFFECTS:

	Est.	S.E.	t val.	d.f.	p
(Intercept)	9.11	0.63	14.36	338.09	0.00
age	-0.02	0.01	-2.38	316.87	0.02
sexM	0.04	0.24	0.18	317.18	0.86
education_level1	-0.31	0.30	-1.06	317.50	0.29
education_level2	-0.68	0.56	-1.21	316.96	0.23
education_level3	-1.51	1.23	-1.23	324.41	0.22
INDEXICU EQ5D	-2.94	0.71	-4.15	317.18	0.00
marital_status1	-0.44	0.43	-1.01	317.51	0.31
life stressor any`1	3.82	0.87	4.37	316.76	0.00
icu_los	-0.02	0.03	-0.63	317.90	0.53
mv1	0.31	0.27	1.14	317.03	0.25
apache_nursing_sheet	0.08	0.03	2.61	317.87	0.01
tmsofa	-0.06	0.05	-1.26	320.40	0.21
benzos any`1	0.38	0.26	1.46	316.90	0.15
TIME2	-2.24	0.18	-12.79	1643.18	0.00
TIME3	-3.66	0.18	-20.89	1643.18	0.00
TIME4	-5.32	0.18	-30.33	1643.18	0.00
TIME5	-6.43	0.18	-36.69	1643.18	0.00
TIME6	-7.24	0.18	-41.06	1644.66	0.00

	Est.	2.5%	97.5%	t val.	d.f.	p
(Intercept)	9.11	7.87	10.36	14.36	338.09	0.00
age	-0.02	-0.04	-0.00	-2.38	316.87	0.02
sexM	0.04	-0.42	0.50	0.18	317.18	0.86
education_level1	-0.31	-0.90	0.27	-1.06	317.50	0.29
education_level2	-0.68	-1.78	0.42	-1.21	316.96	0.23
education_level3	-1.51	-3.91	0.89	-1.23	324.41	0.22

```
INDEXICU EQ5D                 -2.94   -4.32   -1.55    -4.15
317.18   0.00
marital_status1               -0.44   -1.29    0.41    -1.01
317.51   0.31
life stressor any`1            3.82    2.11    5.53     4.37
316.76   0.00
icu_los                       -0.02   -0.09    0.04    -0.63
317.90   0.53
mv1                            0.31   -0.22    0.84     1.14
317.03   0.25
apache_nursing_sheet           0.08    0.02    0.15     2.61
317.87   0.01
tmsofa                        -0.06   -0.16    0.03    -1.26
320.40   0.21
benzos any`1                   0.38   -0.13    0.88     1.46
316.90   0.15
TIME2                         -2.24   -2.59   -1.90   -12.79
1643.18   0.00
TIME3                         -3.66   -4.01   -3.32   -20.89
1643.18   0.00
TIME4                         -5.32   -5.66   -4.97   -30.33
1643.18   0.00
TIME5                         -6.43   -6.78   -6.09   -36.69
1643.18   0.00
TIME6                         -7.24   -7.59   -6.90   -41.06
1644.66   0.00
---------------------------------------------------------------------
--------

---------------------------------------------------------------------
--------
                               Est.    2.5%   97.5%   t val.
d.f.      p
-------------------------- ------- ------- ------- --------
--------- ------
(Intercept)                    7.89    6.86    8.93    14.88
348.24   0.00
age                           -0.33   -0.60   -0.06    -2.38
316.87   0.02
sex                            0.04   -0.42    0.50     0.18
317.18   0.86
education_level1              -0.31   -0.90    0.27    -1.06
317.50   0.29
education_level2              -0.68   -1.78    0.42    -1.21
316.96   0.23
education_level3              -1.51   -3.91    0.89    -1.23
324.41   0.22
INDEXICU EQ5D                 -0.50   -0.74   -0.27    -4.15
317.18   0.00
marital_status                -0.44   -1.29    0.41    -1.01
317.51   0.31
life stressor any              3.82    2.11    5.53     4.37
316.77   0.00
icu_los                       -0.08   -0.35    0.18    -0.63
```

```
317.90   0.53
mv                             0.31   -0.22    0.84     1.14
317.03   0.25
apache_nursing_sheet           0.39    0.10    0.69     2.61
317.87   0.01
tmsofa                        -0.20   -0.50    0.11    -1.26
320.40   0.21
benzos any                     0.38   -0.13    0.88     1.46
316.90   0.15
TIME2                         -2.24   -2.59   -1.90   -12.79
1643.18   0.00
TIME3                         -3.66   -4.01   -3.32   -20.89
1643.18   0.00
TIME4                         -5.32   -5.66   -4.97   -30.33
1643.18   0.00
TIME5                         -6.43   -6.78   -6.09   -36.69
1643.18   0.00
TIME6                         -7.24   -7.59   -6.90   -41.06
1644.66   0.00
--------------------------------------------------------------------
--------

p values calculated using Satterthwaite d.f.

RANDOM EFFECTS:
------------------------------------
  Group      Parameter    Std. Dev.
---------- ------------- -----------
 study_id   (Intercept)     1.81
 Residual                   2.26
------------------------------------

Grouping variables:
----------------------------
  Group     # groups   ICC
---------- ---------- ------
 study_id     331      0.39
----------------------------
```
